# Acquisition and functional consequences of social knowledge in macaques

**DOI:** 10.1098/rsos.160639

**Published:** 2017-02-08

**Authors:** Barbara Tiddi, Eugenia Polizzi di Sorrentino, Julia Fischer, Gabriele Schino

**Affiliations:** 1 Cognitive Ethology Laboratory, German Primate Center, 37077 Göttingen, Germany; 2 Department of Behavioral Ecology, Johann-Friedrich-Blumenbach Institute for Zoology and Anthropology, University of Göttingen, 37077 Göttingen, Germany; 3 Istituto di Scienze e Tecnologie della Cognizione, Consiglio Nazionale delle Ricerche, 00197 Rome, Italy

**Keywords:** social cognition, inter-individual differences, redirected aggression, kinship, primates

## Abstract

To manoeuvre in complex societies, it is beneficial to acquire knowledge about the social relationships existing among group mates, so as to better predict their behaviour. Although such knowledge has been firmly established in a variety of animal taxa, how animals acquire such knowledge, as well as its functional significance, remains poorly understood. In order to understand how primates acquire and use their social knowledge, we studied kin-biased redirected aggression in Japanese macaques (*Macaca fuscata*) relying on a large database of over 15 000 aggressive episodes. Confirming previous research, macaques redirected aggression preferentially to the kin of their aggressor. An analysis that controlled for the rate of affiliation between aggressors and targets of redirection showed that macaques identified the relatives of group mates on the basis of the frequency of their ongoing associations. By contrast, having observed group mates interact with their mother as infants did not increase the monkeys' success in correctly identifying kin relationships among third parties. Inter-individual variation in the successful identification of the kin of aggressors and in redirecting aggression accordingly translated into differences in the amount of aggression received, highlighting a selective advantage for those individuals that were better able to acquire and use social knowledge.

## 1. Introduction

Following kin selection theory, there is a high premium for distinguishing one's own kin from non-kin, and kin recognition is, accordingly, a ubiquitous phenomenon [1,2]. For animals living in complex societies with highly differentiated relationships, it is deemed to be adaptive to extend this recognition to kinship among third parties. Indeed, in the last 35 years, it has become clear that several primate and non-primate species can recognize the kinship ties that exist among other individuals (as well as other types of social relationships among third parties [3–6]). Knowledge of third-party social relationships informs us about two important aspects of animal cognition. First, it shows that some animal species can acquire a non-egocentric understanding of their social world. Second, it shows that they can organize their social knowledge by assigning dyadic relationships to categories such as ‘kin’. Interestingly, primates can also classify dyadic relationships according to multiple criteria simultaneously [7] or even classify them hierarchically [8], while nothing is known about multiple classification in non-primate species.

Despite the progress in the study of animal social cognition, two important aspects remain poorly understood. First, we know very little about how animals acquire their social knowledge and, in particular, how they come to recognize third-party social relationships [9]. Second, we know even less about the extent to which individual variation in social knowledge relates to differences in individual behaviour, and how this may in turn affect fitness [10].

A widely accepted hypothesis about how animals acquire knowledge about the kinship relations of other individuals is that kin are identified on the basis of their ongoing degree of association [11] (Hypothesis 1). For example, Seyfarth & Cheney ([5], pp. 635–636) wrote: ‘it is hard to imagine how a monkey could learn that two other individuals were members of the same matriline except by grouping them together by virtue of their high rates of association’. According to this hypothesis, group-living animals such as primates would monitor the social interactions of group mates and use such information to build and maintain some sort of dyadic ‘index of association’. Such an index would provide a rule of thumb according to which kinship could be estimated. Social behaviour would then be regulated accordingly.

Humans, however, can also rely on the predictable changes in socialization agents that occur across the life cycle. For example, they can remember kin-specific interactions such as parent–infant care for long periods, and thus know that two individuals are related even in the absence of current association. For example, Lieberman *et al.* [12] suggested that humans recognize their siblings (and bias behaviour accordingly) on the basis of the perinatal association of the sibling with their own mother, that is, that they recognize a kinship tie between third parties (one's own sibling and mother) on the basis of past maternal care rather than ongoing association. Obviously, humans also consciously attribute kinship relationships to third parties on the basis of episodic memories of observed maternal care or other early family interactions, and even on the basis of received verbal accounts of such interactions. Considering the complexity of their social interactions and cognitive abilities, it is possible to hypothesize that non-human primates use similar (directly obtained) information and identify the kin of others on the basis of the past observation of kin-specific interactions such as mother–infant care (Hypothesis 2). To our knowledge, these two hypotheses have never been tested, despite their leading to simple and testable predictions. Hypothesis 1 predicts that animals should not distinguish between the kin and the ‘friends’ of others, i.e. between dyads composed of kin and dyads of individuals that happen to show high rates of association in the absence of kinship ties. Hypothesis 2 predicts that animals should in contrast distinguish between the kin and the friends of others, but only if they have had the possibility to observe them as infants.

The evaluation of the fitness consequences of inter-individual variation in cognitive performance has rarely been attempted [6,10,13]. A few studies showed that males that are better at solving ecological problems enjoyed greater female preference (suggesting sexual selection may favour the evolution of higher cognitive abilities; [14,15]) or produced larger clutches [16]. It is more difficult to evaluate the fitness consequences of inter-individual variation in social knowledge, and indeed no study has ever shown that any benefit is directly associated with increased social cognitive performance. The only study that addressed this issue [17] showed that female baboons that scored higher on a personality dimension associated with stronger and longer lasting positive social relationships (themselves associated with increased fitness) were also more responsive in playback experiments involving the understanding of others' social relationships.

Agonistic interactions form an aspect of primate social life that has relevant fitness consequences [18] and in which primates make use of the most complex features of their social cognition. Indeed, the recognition of third-party kin relationships has first been shown in this context ([19], see also [20]). Also, the recruitment of allies during aggressive confrontations has been used to show that primates arrange group mates into a linear hierarchy and thus have a non-egocentric view of the dominance structure of their group [21,22]. In particular, the events following the receipt of aggression constitute an aspect of primate sociality that lends itself to tackling the unresolved issues about social cognition outlined above. On receiving aggression, primates respond by adopting one of several strategies, each leading to complex chains of events [23]. Two of the most common events that follow the receipt of aggression are of interest here: redirected aggression by the victim and renewed aggression by the original aggressor (see electronic supplementary material, figure S1 for a schematic of these events). These are both phenomena that appear to be widespread across the primate order [23,24].

It has been repeatedly shown that redirected aggression is often targeted at the kin of the original aggressor, thus indicating that the victim is aware of the kinship relationship that exists between the original aggressor and the target of redirected aggression [3,25–28]. This social setting thus offers the possibility to investigate the acquisition of social knowledge. Specifically, by evaluating whether monkeys distinguish between the kin and friends of other group members, one can test the two hypotheses outlined above about the role of ongoing and past association in identifying kinship relationships. Furthermore, by evaluating whether redirecting aggression to the kin of the aggressor is associated (either in the short or long term) with a reduction in received aggression, one can also test whether variation in the identification of kin is related to variation in the benefit of reduced received aggression. We addressed these issues relying on a large database of over 15 000 aggressive episodes recorded while studying a group of captive Japanese macaques (*M. fuscata*). We used randomly created control points to compare the behaviour of macaques after receiving aggression with an appropriate control. Control points were created by shifting the timing of observed aggressive episodes, and retained the identities of the aggressors and victims (see details in Material and methods). Control points thus allowed a comparison of the observed versus expected timing of redirected aggression, as well as a comparison of the observed versus expected degree of kinship between aggressors and targets of redirection.

We show that macaques identify the relatives of group mates on the basis of their ongoing frequent association, and that inter-individual differences in the ability to recognize kin translate into differential benefits in terms of reduced received aggression.

## 2. Results

### 2.1. Redirected aggression and its targets

Japanese macaques were much more likely to attack a third individual after receiving aggression than after control points. Overall, aggression to a third party occurred before the end of the observation session in 13.8% of cases after receiving aggression and only in 9.3% of cases after control points (survival analysis: *χ*^2^* *= 205.39, d.f. = 1, *p *< 0.001; electronic supplementary material, figure S3). Thus, redirecting received aggression to a third party was a phenomenon at play in our study group.

**Figure 1.**
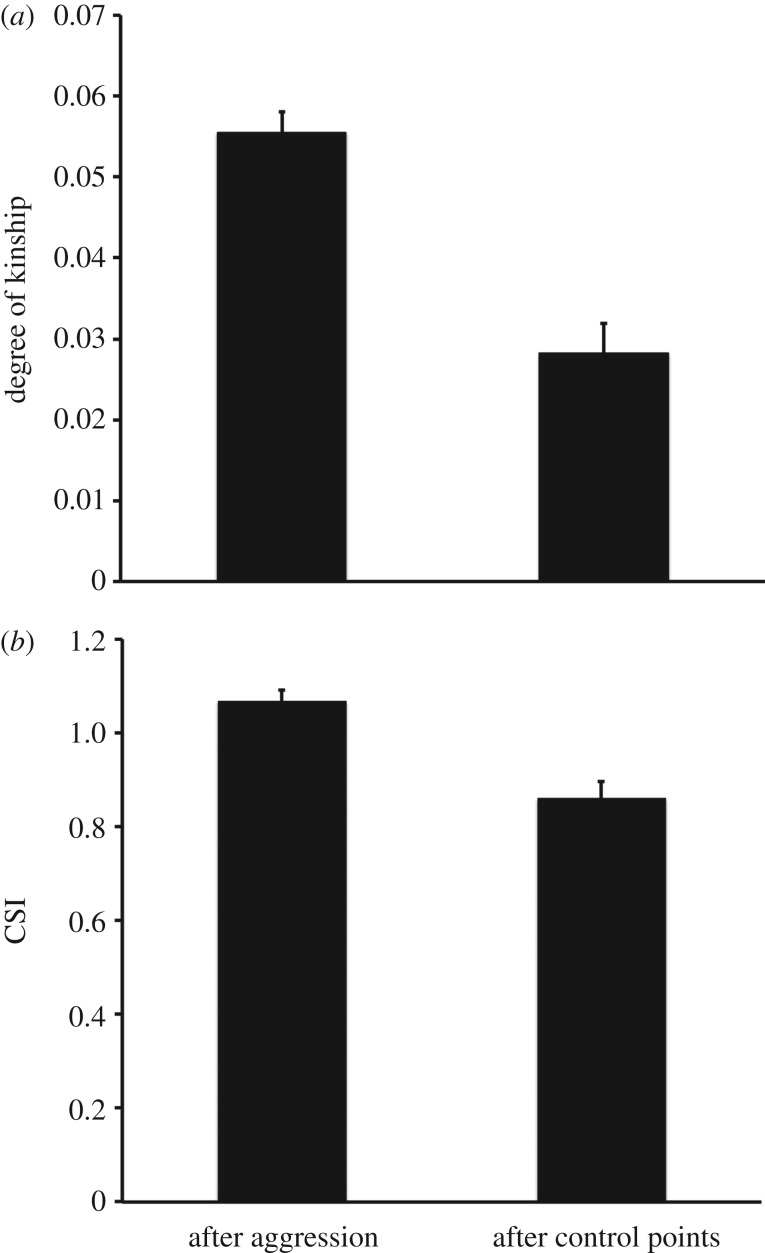
Kinship and affiliation between aggressor and target of redirection. (*a*) Degree of maternal kinship between aggressor and target of redirection after observed aggressive episodes (*N* = 1101) or after control points (*N* = 744). (*b*) CSI between aggressor and target of redirection after observed aggressive episodes (*N* = 1890) or after control points (*N* = 1307). Marginal means and standard errors are shown.

The degree of kinship between the aggressor and the target of redirection was higher than that expected on the basis of control points, showing that macaque victims redirected aggression preferentially to the kin of their aggressors (within-subject linear regression: coeff. = 0.027, *t *= 4.38, d.f. = 56, *p *< 0.001; figure 1*a*).

Excluding all cases in which the target of redirection was a relative of the aggressor, the association score (Composite Sociality Index, CSI, [29]; calculated on the basis of data recorded over the whole year of observation) between the aggressor and the target of redirection was higher than that expected on the basis of control points (coeff. = 0.204, *t* = 3.31, d.f. = 56, *p* = 0.002; figure 1*b*). Thus, along with redirecting aggression to the kin of the aggressor, victims also redirected aggression preferentially to the non-kin ‘friends’ of their aggressors. This held true also when kin were included and the effect of kinship was controlled for (coeff. = 0.629, *t* = 6.79, d.f. = 56, *p* < 0.001).

**Figure 2.**
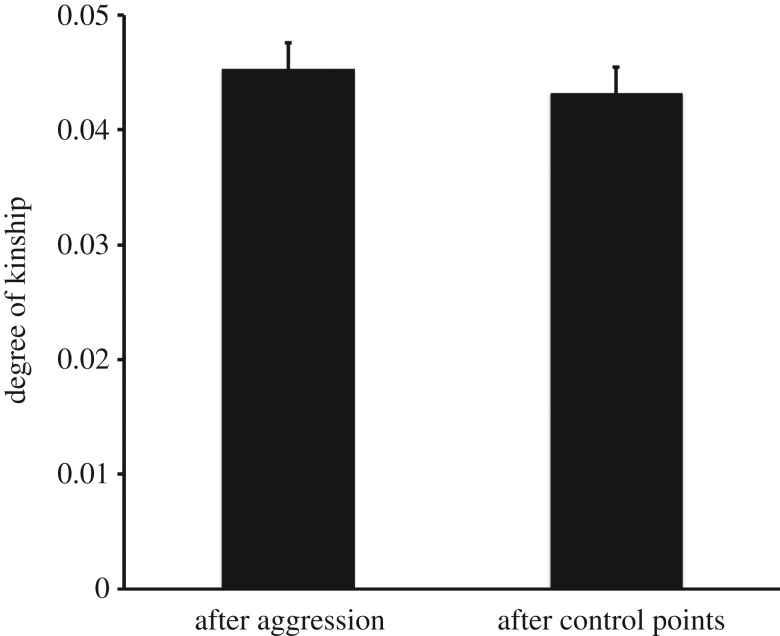
Kinship between aggressor and target of redirection, controlling for affiliation. Degree of maternal kinship between aggressor and target of redirection after observed aggressive episodes (*N* = 1101) or after control points (*N* = 744), controlling for the CSI between aggressor and target of redirection. Marginal means and standard errors are shown.

In order to assess the role of social association in the choice of the target of redirected aggression (i.e. to understand how macaques identified the kin of their aggressors), we tested again whether the degree of kinship between the aggressor and the target of redirection was higher than that expected on the basis of control points, now controlling for the association score (CSI) between the aggressor and the target of redirection. Controlling for CSI, the degree of kinship between the aggressor and the target of redirection was no longer higher than expected (coeff.* *= 0.002, *t *= 0.54, d.f.* *= 56, *p *= 0.588; figure 2). The coefficient obtained in this analysis was also significantly lower than the corresponding coefficient in the analysis above that did not control for CSI (*t *= −12.93, d.f.* *= 54, *p *< 0.001). These results suggest macaques use ongoing overall association rather than actual kinship between third parties in order to choose the target of their redirected aggression, thus supporting Hypothesis 1.

**Figure 3.**
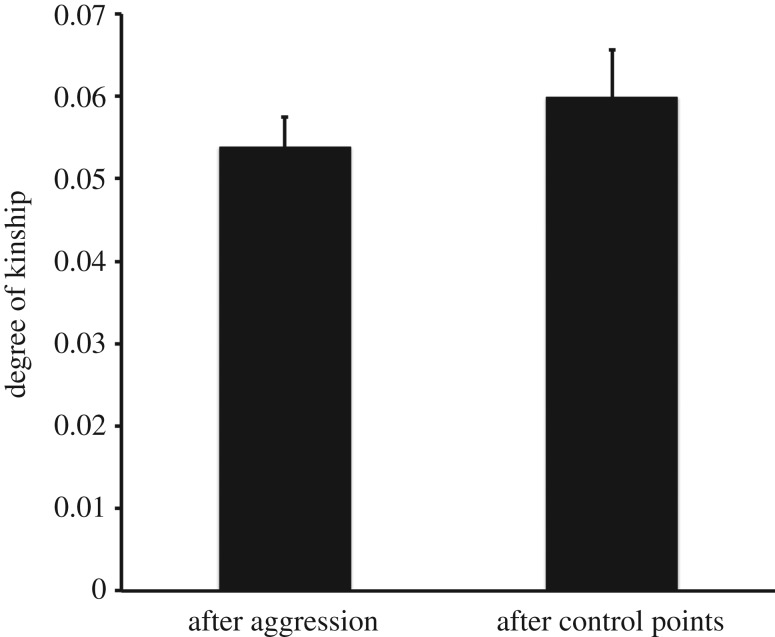
Kinship between aggressor and target of redirection when the victim was older than both, controlling for affiliation. Degree of maternal kinship between aggressor and target of redirection after observed aggressive episodes (*N* = 239) or after control points (*N* = 153), controlling for the CSI between aggressor and target of redirection and including only those aggressive episodes in which the victim was older than both the aggressor and the target of redirection. Marginal means and standard errors are shown.

In order to assess the influence of having observed maternal perinatal associations on the macaque ability to distinguish between the kin and friends of other individuals, we repeated the analysis above including only those aggressive episodes in which the victim was older than the aggressor or older than both the aggressor and the target of redirection. Again, the degree of kinship between the aggressor and the target of redirection was not higher than that expected on the basis of control points when CSI was controlled for (coeff.* *= 0.011, *t *= 1.18, d.f.* *= 42, *p *= 0.245 and coeff.* *= −0.006, *t *= −0.64, d.f.* *= 38, *p *= 0.523, respectively; figure 3). Also, controlling for CSI resulted in significant decreases in the coefficients (*t *= −6.75, d.f.* *= 40, *p *< 0.001 and *t *= −35.26, d.f.* *= 36, *p *< 0.001, respectively). Including only those cases in which the victim was at least 4 years old (and thus beyond the juvenile years) at the time of the aggressor's birth (or of both the aggressor's and the target's birth) did not change the results (coeff.* *= 0.017, *t *= 1.01, d.f.* *= 27, *p *= 0.319 and coeff.* *= −0.009, *t *= −0.63, d.f.* *= 25, *p *= 0.531, respectively). Again, controlling for CSI resulted in significant decreases in the coefficients (*t *= −3.89, d.f.* *= 25, *p *< 0.001 and *t *= −2.87, d.f.* *= 23, *p *= 0.009, respectively). These results show that having observed mother–infant interactions does not improve the macaque ability to distinguish between the kin and friends of their opponent when choosing the target of their redirected aggression, thus contradicting Hypothesis 2.

Summarizing, macaques redirected aggression preferentially to the kin of their aggressor, and identified them on the basis of their ongoing overall association. Macaques also redirected aggression to the non-kin friends of their aggressor. Thus, macaques redirected aggression preferentially towards all individuals showing close association with their aggressors, but did not distinguish the aggressor's kin from the aggressor's friends.

### 2.2. Short-term consequences of redirected aggression

Japanese macaque victims were much more likely to be attacked again by the same aggressor after a first aggressive episode than after control points. Overall, received aggression by the original aggressor occurred again before the end of the observation session in 6.9% of cases after receiving aggression and only in 1.3% of cases after control points (survival analysis: *χ*^2^* *= 631.49, d.f.* *= 1, *p *< 0.001; electronic supplementary material, figure S4). Similarly, victims were more likely to be attacked again by a third individual. Overall, received aggression by a third party occurred before the end of the observation session in 18.5% of cases after receiving aggression and only in 12.4% of cases after control points (survival analysis: *χ*^2^* *= 261.03, d.f.* *= 1, *p *< 0.001).

Redirecting aggression did not reduce the probability of receiving further aggression by the original aggressor (within-subject logistic regression: coeff.* *= 0.173, *z *= 1.21, *p *= 0.226). The effect of redirection on the probability of receiving further aggression by the original aggressor was also not modulated either by the degree of kinship between the aggressor and the target of redirection (coeff.* *= −1.027, *z *= −0.58, *p *= 0.560) or by their association score (excluding all cases in which the target of redirection was a kin of the aggressor) (coeff.* *= 0.104, *z *= 1.65, *p *= 0.100). Redirecting aggression caused an increase in the probability of receiving further aggression by a third party (coeff.* *= 0.405, *z *= 4.75, *p *< 0.001). Overall, redirecting aggression was not associated with any immediate benefit, regardless of the identity of its target.

### 2.3. Long-term consequences of redirected aggression

**Figure 4.**
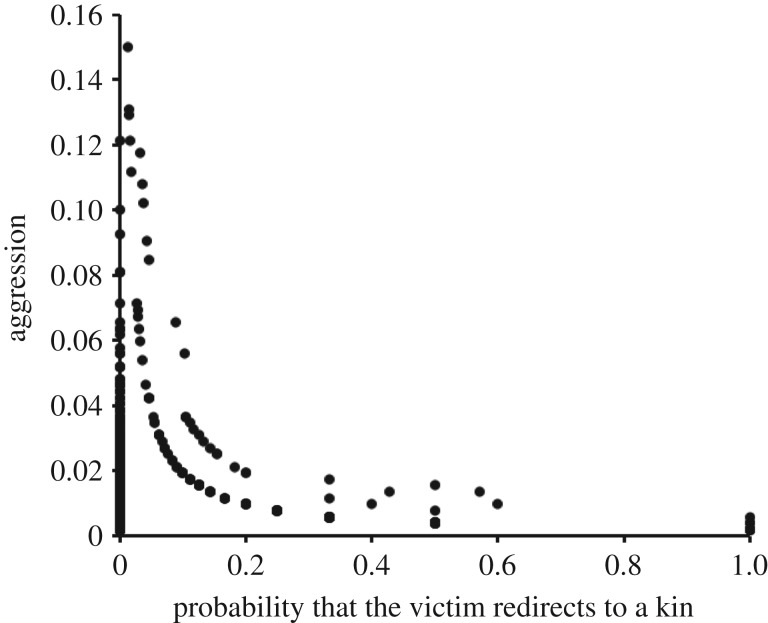
Aggression and victim's redirection to own kin. Aggression given (episodes per hour) in relation to the probability that the victim redirects aggression to one's own kin. Each point represents a different dyad (*N* = 1428).

**Figure 5.**
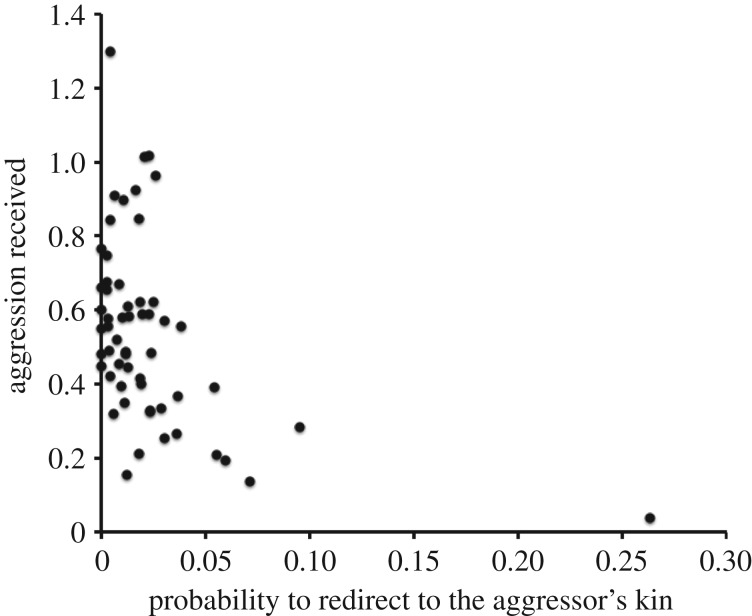
Aggression received and redirection on the aggressor's kin. Aggression received (episodes per hour) in relation to the probability to redirect aggression to the aggressor's kin. Each point represents a different individual (*N* = 57).

Based on dyadic aggression rates recorded over the entire study period, macaques tended to attack less those individuals who more often redirected received aggression to their (that is, the aggressor's) kin (within-subject linear regression: coeff.* *= −1.908, *t *= −2.62, d.f.* *= 40, *p *= 0.012; controlling for rank difference and for sex and age of the victim, and excluding both aggressors with no kin in the group and related aggressor–victim dyads; figure 4). By contrast, macaques did not show less aggression to those individuals who more often redirected received aggression to their friends (coeff.* *= −0.714, *t *= −1.00, d.f.* *= 40, *p *= 0.324). When between-individual, rather than within-individual, variations were analysed, those individuals that redirected aggression more often to the kin of their aggressor received overall less aggression, while redirecting to the friends of the aggressor had no significant effect (controlling for rank, sex and age; coeff.* *= −0.983, *t *= −2.61, d.f.* *= 51, *p *= 0.012 and coeff.* *= −0.232, *t *= −0.43, d.f.* *= 51, *p *= 0.671, respectively; figure 5). These results suggest that redirecting aggression to the kin of the aggressor was effective in reducing aggression received over the long term, while redirecting aggression to its friends was not.

## 3. Discussion

The Japanese macaques we observed in this study redirected aggression preferentially towards the kin of their aggressor, confirming the findings of several previous studies [3,25–28]. In principle, this result could be explained as a simple by-product of the spatial distribution of animals, because kin are likely to be found close to the original aggressor. We think, however, that the ability to recognize kin relationships among third parties cannot be fully explained as a by-product of spatial relationships for at least two reasons. First, this ability has been demonstrated in a variety of species in both wild and captive settings, that is, under variable spatial constraints. Indeed, it has also been demonstrated experimentally in contexts that are fully independent of spatial constraints [11,30]. Second, a previous study on this same social group showed that Japanese macaques are less (*not* more) likely to try to recruit a kin of their opponent during aggressive confrontations despite the presumed higher nearby availability of the kin of the opponent [7]. This showed that the recognition of kin relationships among third parties can easily overcome spatial constraints. Overall, although a role of spatial relationships in explaining kin-biased redirection cannot be completely ruled out, we favour a more cognitively rich interpretation.

Our results suggest macaques identified the kinship relationships of their group mates on the basis of the observation of their ongoing degrees of association. In fact, they also redirected aggression to the non-kin friends of their aggressor and, importantly, did not preferentially target the kin of their aggressor once the ongoing degree of association was controlled for (although lack of information about paternal kinship might have introduced some noise in the data). Furthermore, having had the possibility to observe group mates at an early life stage (i.e. during mother–infant interactions) did not increase the macaque ability to identify kinship relationships independently of ongoing association. These results support Hypothesis 1 over Hypothesis 2, that is, they support the hypothesis macaques identify the kin of others on the basis of their ongoing frequent association rather than on the basis of the past observation of kin-specific interactions. Ours is the first study that explicitly tests alternative hypotheses about how animals come to recognize kinship relationships among third parties, an ability central to the social life of several group-living animals and essential for the management of their social relationships [23].

Interestingly, redirecting aggression to a kin or friend of the aggressor had no detectable short-term consequences, so that macaques could not rely on simple contingencies to identify kinship and friendship relationships (see [28] for different results). At the same time, it is obvious that the simple observation of two individuals' frequent affiliative interactions is not associated with any immediate reward. Categorizing group mates into distinct groups (be they kin or friends) does not seem thus to rely on standard processes of associative learning such as the formation of equivalence classes (*contra* [31]), as these are generally formed on the basis of some history of direct or indirect reinforcement [32].

The preferential redirection of aggression to the kin of the aggressor was associated with long-term positive consequences in terms of reduced rates of aggression received, while redirecting to the friends of the aggressor was not associated with any long-term benefit. In principle, this negative association between aggression and redirection does not provide information on the direction of the underlying causal relations. That is, it is in principle possible either that variation in the behaviour of aggressors (i.e. variable aggression rates) drove variation in the behaviour of victims (variable probability to redirect to the kin of the aggressor), or the reverse. We note, however, that if variation in the behaviour of aggressors had caused variation in the behaviour of victims, then one would logically expect a positive, rather than negative, association. Victims would be expected to increase their redirection to the kin of the most frequent aggressors as a form of revenge. Thus, if variation in the behaviour of aggressors had caused variation in the behaviour of victims, the biological interpretation of the negative association we observed would be difficult. By contrast, a negative relation has an easier biological interpretation if one assumes the opposite direction of causal relations. We, therefore, suggest it was variation in the victims' ability to redirect aggression to the kin of aggressors that drove variation in the behaviour of aggressors.

The differential long-term response of aggressors to redirection targeted at their kin or at their friends constitutes a selective pressure on victims of aggression for distinguishing between the kin and friends of aggressors. Despite the possible role of this selective pressure, macaques did not adopt a more sophisticated strategy (identifying kin on the basis of their past history of interactions) and instead adopted a cognitively simpler strategy (identifying kin on the basis of their ongoing degree of association). Macaques were thus forced to adopt a suboptimal social strategy. However, as ongoing association does provide a reliable index of kinship (see the electronic supplementary material), this suboptimal strategy probably nevertheless constitutes an acceptable rule of thumb (see also [33]).

Our results on the long-term consequences of redirected aggression emphasize the importance of analysing inter-individual differences in the ability to make use of social knowledge [10]. Macaques attacked less often those group mates that more often redirected aggression to their kin, and individuals that redirected aggression more often to the kin of their aggressors received overall less aggression. Thus, the ability to correctly identify kin had positive functional consequences that provide a rare example of the benefit of social knowledge. It should be noted, however, that the benefits we demonstrated are only indirectly related to actual fitness benefits. We have shown that the ability to correctly use social knowledge is associated with patterns of aggression that are themselves known to be associated with health and reproductive benefits [18,34]. However, we still lack direct evidence linking variation in social knowledge with variation in fitness. This is in contrast to the good evidence relating the ability to form strong and enduring social bonds with actual fitness benefits [35–39]. Thus, identifying fitness benefits associated with social knowledge remains a priority for testing the social intelligence hypothesis [5].

Although they did not identify the kin of others independently of their degree of affiliation, macaques were apparently well aware of the difference between their own kin and friends. They refrained from attacking those individuals that frequently redirected aggression to their kin (thus avoiding the consequent indirect fitness costs) but did not refrain from attacking those individuals that frequently redirected aggression to their friends, because no fitness costs are associated with that. These results suggest macaques experienced social relationships with their kin as different from that with friends and suggest the ‘functional equivalence’ between kin and friends ([40–43] see also [44]) may not be entirely applicable to the social life of macaques. This is clearly an aspect that will require further investigation.

The development of cognitive ethology over the last three decades has provided a wealth of information about the social knowledge of primates and other animals, and has shown their impressive capabilities of categorization of this knowledge. We still know very little, however, about how this social knowledge is acquired and updated. Similarly, testing functional hypotheses about the evolution of social cognition will require a better understanding of the benefits associated with social knowledge. Our study provides a first attempt to address these issues and will, hopefully, stimulate further research along these lines.

## 4. Material and methods

### 4.1. Subjects and housing

We studied a group of Japanese macaques living in the Rome zoo (Bioparco). The group was formed by 57 monkeys (11 mature males, 23 mature females and 23 immatures) that descended from a natural group of macaques captured as a whole in Takasakiyama, Japan, and transferred to the Rome zoo in 1977. Macaques were housed in a 700 m^2^ outdoor enclosure connected to indoors quarters.

The group has been studied extensively since its arrival in Rome [45], and genealogical relationships were derived from demographic records. Details on the group history and housing conditions can be found in [46,47].

### 4.2. Data collection

Data were collected between July 2003 and July 2004 by two observers working simultaneously. Monitoring the entire enclosure, and using a combination of the ‘focal group’ and ‘complete record’ observation techniques [48], the two observers recorded the timing and the individuals involved in every episode of agonistic behaviour (threats, chases and physical assaults, as defined in [49]). At the end of each 30 min observation session a group scan was carried out, recording all dyads involved in allogrooming and sitting in passive contact. A total of 519.3 h of complete record observation and 1018 group scans were completed.

This study was originally conceived with different aims (see [7,50]). The observers can, therefore, be considered as blind in relation to the hypotheses tested in this study.

### 4.3. Data analysis

We calculated dyadic scores for rates of aggression given and received, proportion of time spent grooming and sitting in contact, and probability of coalition formation. We used dyadic scores of grooming, sit in contact and coalitions collected over the entire study period to generate dyadic values of the CSI [29]. These were used as a measure of the ongoing degree of association. Degrees of maternal kinship were derived from demographic records. Kinship and CSI were entered as continuous variables into statistical analyses. When, however, we had to categorize dyads as kin or non-kin or as friend or non-friend (for example, to include or exclude some dyads from analysis), we considered as kin those dyads with *r* ≥ 0.125 and as friends those non-kin dyads with a CSI larger than the group median.

We adopt the following terms (see also electronic supplementary material, figure S1): the aggressor is the individual that initiated an aggressive episode; the victim is the individual that received the aggressive episode; the target of redirection is the individual that received the first aggression by the original victim following the original aggression (or a control point). Most analyses were based on a comparison of the behaviour of macaques following the receipt of aggression or random control points. Random control points were generated by shifting the timing of each observed aggressive episode by 10 observation sessions (i.e. in each 30 min observation session, control points had the same timing of the real aggressive episodes observed in the 10th observation session preceding it). Control points retained the identities of the original aggressor and victim. In this way, we obtained a sample of control points that was comparable with that of the observed aggressive episodes both in total size per each victim and aggressor and in its seasonal fluctuations. This method follows [48] and is equivalent to the post-conflict/matched control method commonly used in studies of post-conflict behaviour [51].

We used survival analysis (the Peto–Peto test) to test for the occurrence of redirected aggression and of renewed aggression. Individual aggressive episodes were the unit of analysis, and we inserted the victim identity as a stratification variable in order to avoid pseudoreplication and obtain within-subject analyses. In order to test for redirected aggression, we compared the time elapsed between the receipt of aggression and the first aggressive episode by the victim with that between control points and the first aggressive episode by the victim. We used the same strategy when testing for renewed aggression, and compared the time elapsed between the receipt of the original aggression and the receipt of another aggressive episode from the same (or from another) aggressor with the time elapsed between random control points and the receipt of aggression.

We used within-subject (fixed-effect) linear regressions with robust standard errors [52] to compare the degree of kinship and/or CSI between the aggressor and the target of redirection after observed aggressive episodes and after control points (remember that control points were associated with the identities of a victim and an aggressor). Individual aggressive episodes were our unit of analysis. We followed the methods proposed by [53] for comparing the coefficients of analyses that controlled or did not control for the effect of CSI.

We used conditional within-subject (fixed-effect) logistic regressions [52] to compare the probability of renewed aggression by the original aggressor (or by a third party) after redirection (i.e. aggression by the original victim to a third party) and in the absence of redirection. A similar analysis evaluated whether the probability of renewed aggression was modulated by the degree of kinship or by the CSI between the original aggressor and the target of redirection. In these analyses, we entered the natural logarithm of the time between redirection (or, in the absence of redirection, the mean time to redirection) and the end of the observation session as an offset variable. Individual aggressive episodes were our unit of analysis.

We used a within-subject (fixed-effect) linear regression with robust standard errors to evaluate the relations between the rate of aggression given and the probability that the victim would redirect aggression to the aggressor's kin or friends. Dyadic scores were the unit of analysis. Finally, we used a linear regression with robust standard errors to evaluate the relations between the rate of total aggression received and the overall probability to redirect aggression on an aggressor's kin or friend. Individuals were the unit of analysis.

All analyses were run using Stata 14.1 [54]. Complete regression tables, including sample sizes, are shown in the electronic supplementary material.

## Supplementary Material

ESM provide information about supplementary methods and results, along with tables from the statistical analyses described in the main manuscript.

## Data accessibility

Raw data are available in the Dryad Digital Repository: http://dx.doi.org/10.5061/dryad.6q442 [55].
