# Supplementary material for: Acquisition and functional consequences of social knowledge in macaques
Source: R Soc Open Sci. 2017 Feb 8;4(2):160639. doi: 10.1098/rsos.160639 (PMC5367287; doi:10.1098/rsos.160639)
Supplement: ESM provide information about supplementary methods and results, along with tables from the statistical analyses described in the main manuscript. [file rsos160639supp1.docx]

Supplementary Materials for

**Acquisition and functional consequences of social knowledge in macaques**

Barbara Tiddi, Eugenia Polizzi di Sorrentino, Julia Fischer and Gabriele Schino

SUPPLEMENTARY METHODS


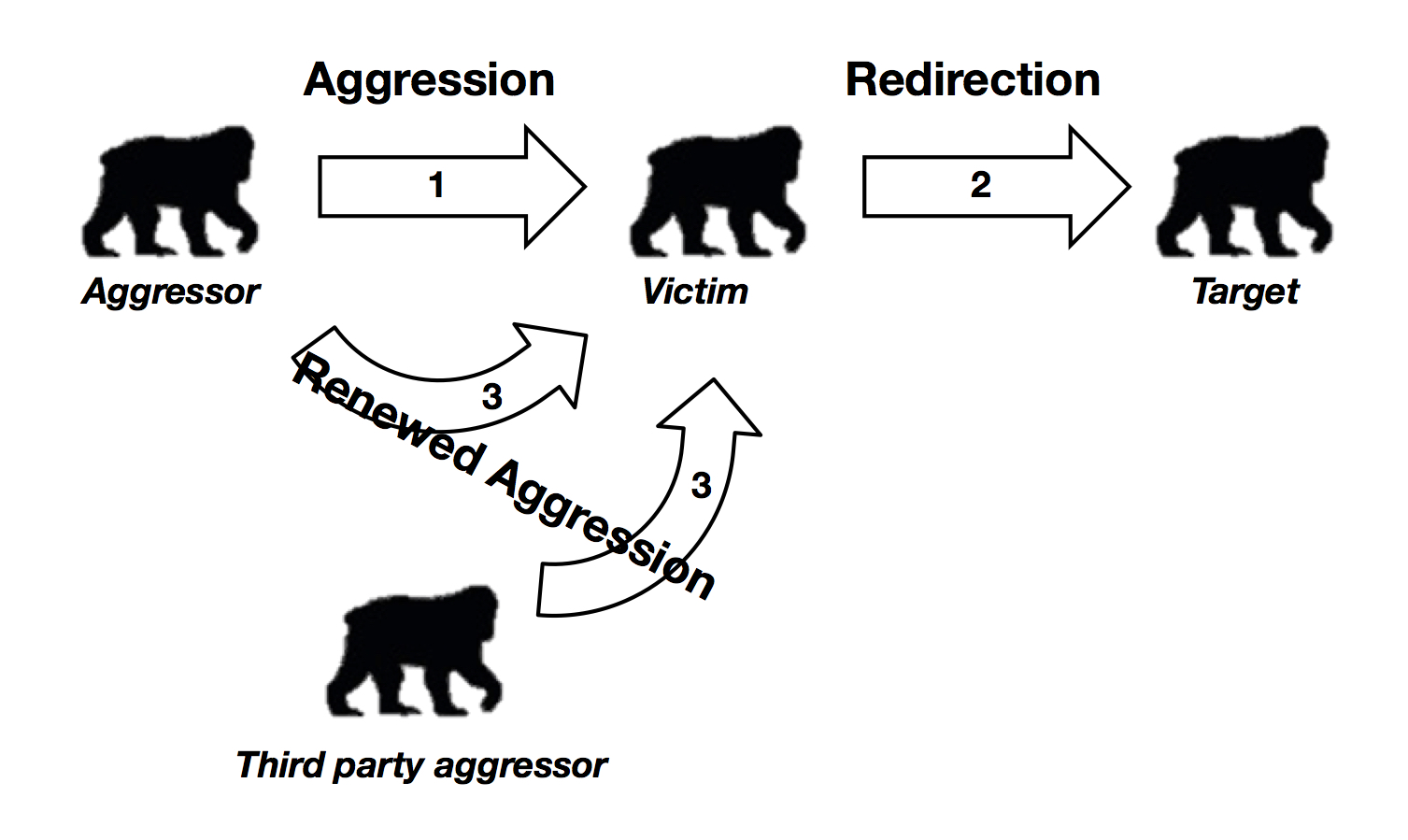


**Figure S1.** Schematic representation of aggressive events analysed in this study. Numbers in the arrows indicate the sequence of events.

SUPPLEMENTARY RESULTS

Kinship and Friendship

Macaques in our group had on average 4.46 relatives in the group (i.e., individuals with a degree of kinship r≥0.125) and 24.95 "friends" (i.e., nonkin individuals with a CSI>0.275, the group median). Mean CSI for kin was 4.964, mean CSI for friends was 1.305. Unsurprisingly, CSI was positively associated with kinship (within-subject linear regression with robust standard errors: coeff.=24.215, t=8.15, df=56, P<0.001; Fig. S2).

**Figure S2.** Composite Sociality Index (CSI) in relation to degree of maternal kinship (N=3192).

Events following aggression

**Figure S3.** Time course of redirected aggression. Nelson-Aalen cumulative hazard of the first aggression directed by the victim to a third party after receiving aggression (N=15863) or after control points (N=15863)

**Figure S4.** Time course of renewed aggression. Nelson-Aalen cumulative hazard of the first aggression received by the victim from the original aggressor after receiving aggression (N=15863) or after control points (N=15863).

Details of statistical analyses

Below are reported the complete regression tables relative to all analyses included in the main text. Tables include information on sample sizes and on all dependent and independent variables inserted into analyses.

**Dependent var.:** Time to redirected aggression

**Events observed Events expected**

After aggression 2185 1863.69

After control points 1447 1768.31

𝜒^2^=205.39, df=1, P<0.0001

**Table S1.** Stratified Peto-Peto test for equality of survivor functions comparing redirected aggression following received aggression or control points (N=31402 observations).

**Dependent var**.: Degree of kinship between aggressor and target of redirection

**Indep. var. Coeff. t df P**

Type of obs. 0.027 4.38 56 <0.001

Intercept 0.001 0.09 56 0.928

**Table S2.** Within-subject (fixed effect) linear regression with robust standard errors relating the degree of kinship between aggressor and target of redirection to the type of observation (following received aggression or control points) (N=1845 observations; excluding aggressors with no kin and observations in which the aggressor was a kin of the victim).

**Dependent var**.: Composite Sociality Index (CSI) between aggressor and target of redirection

**Indep. var. Coeff. t df P**

Type of obs. 0.207 3.29 56 0.002

Intercept 0.653 6.52 56 <0.001

**Table S3.** Within-subject (fixed effect) linear regression with robust standard errors relating the Composite Sociality Index (CSI) between aggressor and target of redirection to the type of observation (following received aggression or control points) (N=3197 observations; excluding observations in which the target was a kin of the aggressor).

**Dependent var**.: Composite Sociality Index (CSI) between aggressor and target of redirection

**Indep. var. Coeff. t df P**

Type of obs. 0.629 6.79 57 <0.001

Kinship 35.918 11.20 57 <0.001

Intercept -0.273 -1.32 57 0.192

**Table S4.** Within-subject (fixed effect) linear regression with robust standard errors relating the Composite Sociality Index (CSI) between aggressor and target of redirection to the type of observation (following received aggression or control points) (N=3632 observations), controlling for the degree of kinship between aggressor and target of redirection.

**Dependent var**.: Degree of kinship between aggressor and target of redirection

**Indep. var. Coeff. t df P**

Type of obs. 0.002 0.54 56 0.588

CSI 0.137 19.92 56 <0.001

Intercept 0.134 2.16 56 0.035

**Table S5.** Within-subject (fixed effect) linear regression with robust standard errors relating the degree of kinship between aggressor and target of redirection to the type of observation (following received aggression or control points), controlling for the Composite Sociality Index (CSI) between aggressor and target of redirection (N=1845 observations; excluding aggressors with no kin and observations in which the aggressor was a kin of the victim).

**Dependent var**.: Degree of kinship between aggressor and target of redirection

**Indep. var. Coeff. t df P**

Type of obs. 0.011 1.18 42 0.245

CSI 0.014 11.25 42 <0.001

Intercept 0.007 0.43 42 0.669

**Table S6.** Within-subject (fixed effect) linear regression with robust standard errors relating the degree of kinship between aggressor and target of redirection to the type of observation (following received aggression or control points), controlling for the Composite Sociality Index (CSI) between aggressor and target of redirection (N=480 observations; excluding aggressors with no kin, observations in which the aggressor was a kin of the victim and observations in which the aggressor was not younger than the victim).

**Dependent var**.: Degree of kinship between aggressor and target of redirection

**Indep. var. Coeff. t df P**

Type of obs. -0.006 -0.64 38 0.523

CSI 0.014 11.05 38 <0.001

Intercept 0.029 1.92 38 0.063

**Table S7.** Within-subject (fixed effect) linear regression with robust standard errors relating the degree of kinship between aggressor and target of redirection to the type of observation (following received aggression or control points), controlling for the Composite Sociality Index (CSI) between aggressor and target of redirection (N=392 observations; excluding aggressors with no kin, observations in which the aggressor was a kin of the victim and observations in which the aggressor and the target were not both younger than the victim).

**Dependent var**.: Degree of kinship between aggressor and target of redirection

**Indep. var. Coeff. t df P**

Type of obs. 0.017 1.01 27 0.319

CSI 0.013 10.65 27 <0.001

Intercept -0.005 -0.19 27 0.851

**Table S8.** Within-subject (fixed effect) linear regression with robust standard errors relating the degree of kinship between aggressor and target of redirection to the type of observation (following received aggression or control points), controlling for the Composite Sociality Index (CSI) between aggressor and target of redirection (N=241 observations; excluding aggressors with no kin, observations in which the aggressor was a kin of the victim and observations in which the victim was not at least 4-year-old at the time of the aggressor's birth).

**Dependent var**.: Degree of kinship between aggressor and target of redirection

**Indep. var. Coeff. t df P**

Type of obs. -0.009 -0.63 25 0.531

CSI 0.013 10.61 25 <0.001

Intercept 0.029 1.21 25 0.239

**Table S9.** Within-subject (fixed effect) linear regression with robust standard errors relating the degree of kinship between aggressor and target of redirection to the type of observation (following received aggression or control points), controlling for the Composite Sociality Index (CSI) between aggressor and target of redirection (N=193 observations; excluding aggressors with no kin, observations in which the aggressor was a kin of the victim and observations in which the victim was not at least 4-year-old at the time of both the aggressor's and the target's birth)

**Dependent var.:** Time to renewed aggression by the original aggressor

**Events observed Events expected**

After aggression 1088 637.84

After control points 204 654.16

𝜒^2^=631.49, df=1, P<0.0001

**Table S10.** Stratified Peto-Peto test for equality of survivor functions comparing renewed aggression received by the victim from the original aggressor following received aggression or control points (N=31402 observations).

**Dependent var.:** Time to renewed aggression by a third party

**Events observed Events expected**

After aggression 2934 2383.96

After control points 1298 2478.96

𝜒^2^=261.03, df=1, P<0.0001

**Table S11.** Stratified Peto-Peto test for equality of survivor functions comparing renewed aggression received by the victim from a third party following received aggression or control points (N=31402 observations).

**Dependent var**.: Occurrence of renewed aggression by the original aggressor (presence/absence)

**Indep. var. Coeff. z P**

Redirection 0.173 1.21 0.226

**Table S12.** Within-subject (fixed effect) conditional logistic regression relating the occurrence of renewed aggression by the original aggressor to the occurrence of redirected aggression by the victim (presence/absence) (N=12199 observations).

**Dependent var**.: Occurrence of renewed aggression by the original aggressor (presence/absence)

**Indep. var. Coeff. z P**

Kinship -1.027 -0.58 0.560

**Table S13.** Within-subject (fixed effect) conditional logistic regression relating the occurrence of renewed aggression by the original aggressor to the degree of kinship between aggressor and target of redirection (N=405 observations; excluding aggressors with no kin and observations in which the aggressor was a kin of the victim).

**Dependent var**.: Occurrence of renewed aggression by the original aggressor (presence/absence)

**Indep. var. Coeff. z P**

CSI 0.104 1.65 0.100

**Table S14.** Within-subject (fixed effect) conditional logistic regression relating the occurrence of renewed aggression by the original aggressor to the Composite Sociality Index (CSI) between aggressor and target of redirection(N=983 observations; excluding observations in which the target was a kin of the aggressor).

**Dependent var**.: Occurrence of renewed aggression by a third party (presence/absence)

**Indep. var. Coeff. z P**

Redirection 0.405 4.75 <0.001

**Table S15.** Within-subject (fixed effect) conditional logistic regression relating the occurrence of renewed aggression by a third party to the occurrence of redirected aggression by the victim (presence/absence) (N=11389 observations).

**Dependent var**.: Dyadic rates of aggression given (calculated over the entire study period)

**Indep. var. Coeff. t df P**

Prob. redir. on kin -1.773 -2.51 40 0.016

Prob. redir. on friend -1.338 -1.50 40 0.140

Rank difference 0.048 1.57 40 0.124

Intercept 5.934 19.11 40 <0.001

**Table S16.** Within-subject (fixed effect) linear regression with robust standard errors relating the rates of aggression given to the probability that the victim redirects aggression on a kin or "friend" of the subject, controlling for rank difference (N=1428 dyads; excluding sujects with no kin and kin dyads).

**Dependent var**.: Individual rates of aggression received (calculated over the entire study period)

**Indep. var. Coeff. t df P**

Prob. redir. on kin -1.058 -2.92 53 0.005

Prob. redir. on friend -0.892 -1.70 53 0.096

Rank 0.003 0.74 53 0.462

Intercept 0.573 3.74 53 <0.001

**Table S17.** Linear regression with robust standard errors relating the rates of aggression received to the probability that the subject redirects aggression on a kin or "friend" of the aggressor, controlling for the subject's rank (N=57 individuals).
